# Abnormal attentional bias in individuals with suicidal ideation during an emotional Stroop task: an event-related potential study

**DOI:** 10.3389/fpsyt.2023.1118602

**Published:** 2023-08-22

**Authors:** Yiwei Sun, Moxin Duan, Li An, Shuang Liu, Dong Ming

**Affiliations:** ^1^Academy of Medical Engineering and Translational Medicine, Tianjin University, Tianjin, China; ^2^School of Education, Tianjin University, Tianjin, China; ^3^Department of Biomedical Engineering, College of Precision Instruments and Optoelectronics Engineering, Tianjin University, Tianjin, China

**Keywords:** attentional bias, suicidal ideation, electroencephalography, event-related potentials, emotional Stroop task

## Abstract

**Introduction:**

There is increasing evidence that suicidal individuals exhibit an attentional bias toward negative or suicide-related stimuli, but the underlying neural mechanism remains unclear. This study aimed to investigate the neural mechanism of attentional bias toward emotional stimuli using a modified emotional Stroop task (EST) and to further explore the influencing factor of abnormal attention processing by identifying whether mental disorders or suicidal ideation contributes to attention processing disruptions.

**Methods:**

Fourteen students with suicidal ideation and mental disorders (SIMDs), sixteen students with suicidal ideation but no mental disorders (SINMDs), and fourteen sex- and age-matched healthy controls (HCs) were recruited. Moreover, 64-channel electroencephalography (EEG) data and behavioral responses were recorded simultaneously during the EST. Participants were instructed to respond to the ink color for various types of words (positive, neutral, negative, and suicide) while ignoring their meanings. Event-related potentials (ERPs) were analyzed to evaluate attention to the stimuli. Spearman correlations between clinical psychological assessment scales and ERP signatures were analyzed to determine the risk factors for suicide.

**Results:**

The results showed that the SIMD group exhibited longer early posterior negativity (EPN) latency compared to the SINMD and HC groups, indicating that early attention processing was affected during the EST, and the automatic and rapid processing of emotional information decreased. Furthermore, P300 latency for positive words was positively correlated with current suicidal ideation in the SINMD group, suggesting that delayed responses or additional processing to positive information may lead individuals with suicidal ideation to an incorrect interpretation of external events.

**Conclusions:**

Generally, our findings suggest that the neural characteristics of the SIMD group differed from those of the SINMD and HC groups. EPN latency and P300 latency during the EST may be suicide-related neurophysiological indicators. These results provide neurophysiological signatures of suicidal behavior.

## 1. Introduction

Suicide has become a serious global public health issue. According to a World Health Organization survey conducted from 2000 to 2019, an estimated 700,000 individuals worldwide die by suicide annually, and suicide become the fourth leading cause of death in young people aged 15–29 years (1). There are likely to be 20 suicide attempts for every suicide (2). However, our ability to understand, predict, and prevent suicide remains inadequate (3). Suicidal behavior is a continuous spectrum of behaviors, mainly including suicidal ideation, suicide attempt, and suicide death (4). Suicidal ideation, the psychological activity in the early stage of suicide, refers to thoughts of self-harm or death without behaviors that threaten one’s survival (5). During the evolution of psychopathology, patients may move from one type of suicidal behavior to another, and the transition from suicidal ideation to suicide attempt is a common path (6). Suicide attempt is a kind of self-harming behavior with a certain degree of suicidal intention, which may lead to the serious consequence of suicide death (7). One study has shown that suicidal ideation is the most sensitive predictor of suicide attempt (8). The occurrence of suicidal ideation may predict future suicide attempts and even suicide death. Therefore, a better understanding of the risk factors of suicide, particularly in individuals with suicidal ideation, is useful for the early identification of suicide, so as to reduce the incidence of suicide attempts and death.

The traditional assessments of suicide primarily depend on clinical interviews and self-report. However, due to the sensitivity and stigma surrounding suicide, people tend to deny or conceal suicidal thoughts and experiences, or avoid discussing this topic with others (9). This highlights the significance of developing alternative measures for identifying individuals with suicidal thoughts or behaviors.

Several cognitive deficits have been reported in suicidal individuals, including attention deficits (6, 10, 11), impaired memory (12, 13), and decision-making impairments (14, 15). A cognitive model for suicide suggests that individuals with suicidal behaviors show attentional bias toward suicide-related stimuli (e.g., the word “suicide”), possibly due to the activation of suicide schemas. These schemas are relatively stable and durable cognitive templates rooted in the early experience of suicidal individuals, helping people integrate and make sense of stimuli they encounter (16). Suicidal individuals cannot correctly attribute value to external events and often have negative emotions when interpreting social environments, which may activate suicidal behaviors under stress and despair (17). According to the schema theory, once the stimulus is consistent with the schema, the processing of information is easier. Therefore, suicidal individuals preferentially focus on suicide-related stimuli and have difficulty disengaging from these stimuli and allocating attention to other goals (18).

Attentional bias can be measured by an emotional Stroop task (EST) (19–22), during which participants are instructed to respond to the ink color of emotional words. In the EST, emotional interference results from an attentional bias toward emotional stimuli, which manifests as poor performance on the color-naming task (e.g., lower response accuracy and longer reaction time to emotional versus neutral words) (21). Moreover, the emotional interference may be amplified when the meaning of the word is related to the nature of the participant’s psychopathology (19). Some EST studies that included suicide-related stimuli found that compared to non-suicide attempters, suicide attempters showed attentional bias to suicide-related stimuli, exhibited by longer reaction times (23–25). Additional research is needed to explore the characteristics as well as the underlying neural mechanism of attentional bias in individuals with suicidal ideation.

Attention processing occurs within several hundred milliseconds. It is typically studied using methods with high temporal resolution, such as electroencephalography (EEG), to investigate rapid changes in cognitive processes. Event-related potentials (ERPs) are useful to study attention, as they can reflect cognitive processing at the millisecond level (23, 26). Two ERP components, the early posterior negativity (EPN) and P300, can be found in various stages of attention processing during the EST. The EPN is a negative-going deflection over parieto-occipital sites, peaking between 200 and 300 ms after stimulus presentation. It is related to early attentional selection and reflects automatic processing of emotional stimuli (27–31). Previous EST studies have found that the EPN amplitude induced by emotional words or pictures (positive and negative) was larger (i.e., more negative) than that induced by neutral words or pictures (32–35). In a study classifying facial and non-facial stimuli with emotional information, Xin et al. (31) found that EPN was significantly delayed and lower (i.e., more positive) in major depressive disorder (MDD) patients compared to the control group. Their results revealed the dysfunction of early attentional processing of salient emotional faces in MDD. Tavakoli et al. (23) employed an EST including suicide-related words in suicide attempters aged 13–17 but found no significant difference in EPN between groups or across word categories. In addition, no studies have analyzed EPN in suicidal individuals. Another ERP component, the P300 (also called the P3) is a positive peak that occurs approximately 300–500 ms following stimulus onset, with maximum amplitude over centro-parietal electrode sites. The P3 is associated with cognitive activities such as attention, decision-making, and memory (36–40). The results of previous EST studies showed that negative stimuli elicited larger P3 amplitude than neutral stimuli (32, 41). Thomas et al. (42) found larger P3 amplitude to threatening words than neutral words in a modified Stroop task that asked participants to determine whether each word was threatening; however, when the participants were instructed to name the color of the words, P3 amplitude differences between the two word types decreased. Tavakoli et al. (23) observed double-P3 in suicidality, with the early-P3 appearing at approximately 300 ms and the late-P3 appearing at approximately 450–500 ms. Compared to healthy controls (HCs), early- and late-P3 were significantly lower, and late-P3 latency was longer in suicide attempters, suggesting that suicide attempters may adopt different cognitive strategies for the color-naming task.

Generally, although few ERP studies have been conducted on individuals with suicidal behaviors using an EST, previous studies have found that individuals with suicidal behaviors exhibited attentional bias toward suicide and/or negative stimuli compared to HCs (17, 23, 43). However, there are several problems that need to be addressed. First, the behavioral responses and neural mechanism of attentional bias in individuals with suicidal behaviors remain unclear. Second, most samples in previous studies were patients with suicidal ideation or suicide attempts with comorbid mental disorders, such as depression and anxiety, leading to difficulty in determining whether attention deficits are a result of suicide behaviors or mental disorders.

This study aimed to investigate behavioral responses and the neural mechanism of attentional bias toward emotional stimuli using a modified EST in a group with suicidal ideation and to further explore the influencing factor of abnormal attention processing. In other words, the current study sought to identify whether mental disorders or suicidal ideation contributes to attention processing disruptions. We hypothesized that, compared to HCs, individuals with suicidal ideation as well as mental disorders (SIMDs) and individuals with suicidal ideation but no mental disorders (SINMDs) would show abnormal attentional bias in response to suicide and/or negative stimuli. Further, we expected that there would be no significant difference in attentional bias between SINMDs and SIMDs, or that SIMDs would show more severe attentional bias than SINMDs due to mental disorders. These analyses have the potential to shed new light on task performance and the underlying neural mechanism of individuals with suicidal ideation during attentional bias.

## 2. Materials and methods

### 2.1. Participants

We recruited participants with current or lifetime history of suicidal ideation from the mental health education center at a university in Tianjin and HCs from general university students through fliers and advertisements. All participants were right-handed undergraduate or graduate students, with age ranging from 17 to 24 years. They had normal or corrected-to-normal vision. The exclusion criteria were as follows: (1) history of head injury or diagnosed with chronic somatic diseases, (2) color blindness and color weakness, (3) an inability to read and understand the materials given to him/her, and (4) suicide attempt history. A Chinese version of the Mini-International Neuropsychiatric Interview (MINI) 5.0 (44), a structured interview tool with good reliability and validity, was used in our study to screen mental disorders and history of suicidal behaviors. It was mainly used to screen and diagnose 16 axis I mental disorders and one personality disorder in the Diagnostic and Statistical Manual of Mental Disorders, Fourth Edition (DSM-IV) and the International Statistical Classification of Mental Disorders (ICD-10). We excluded one color-impaired student, two left-handed students, and several students with histories of suicide attempts. Another student was unable to complete the experiment because of his/her social phobia. Eventually, we collected data from 14 SIMDs, 16 SINMDs, and 14 sex- and age-matched HCs. Recruiting participants with suicidal ideation posed inherent challenges, making it difficult to obtain an ample sample size. The study’s sample size was determined based on similar studies (23, 34). Nevertheless, according to MorePower 6.0, the current study is considered underpowered. Consequently, the limited sample size may impact the statistical power and generalizability of the findings, making it essential to interpret the results cautiously.

The study was approved by the Ethics Committee of Tianjin University, China. Prior to the study, all participants signed the written informed consent. It should be noted that there was one 17-year-old first-year university student in the study whose parents were not local. As a result, only verbal consent was obtained from his/her guardian. All participants received remuneration and a gift box including a gel pen, notebook, and key chain for their participation. The demographic and clinical psychological characteristics of all groups are listed in Table 1.

**Table 1:** Demographic and clinical psychological characteristics of the HCs, SINMDs, and SIMDs.

	SIMDs	SINMDs	HCs	Statistics	*p* value	Post-hoc
Demographics						
Sample size	14	16	14			
Sex (male/female)	6/8	5/11	7/7	χ^2^ = 1.118	0.572	/
Age (year)	20.36 (2.44)	18.94 (1.57)	21.00 (2.11)	H = 5.092	0.078	/
Clinical scales						
BDI score	14.71 (8.35)	7.19 (6.32)	2.93 (2.90)	H = 12.138	0.002	SIMD>HC
SAS score	47.93 (13.93)	38.75 (7.46)	36.21(5.38)	F = 4.292	0.025	SIMD>SINMD, HC
SBQ-R score	8.57 (3.67)	6.19 (1.87)	3.14 (0.36)	H = 29.483	<0.001	SIMD, SINMD>HC
BSI-C score	2.00 (2.51)	0.19 (0.54)	0.14 (0.54)	H = 10.168	0.006	SIMD>SINMD, HC
BSI-W score	5.71 (2.81)	4.31 (2.09)	0.21 (0.58)	H = 28.315	<0.001	SIMD, SINMD>HC

Data are presented as mean (SD). SIMDs, individuals with suicidal ideation as well as mental disorders; SINMDs, individuals with suicidal ideation but no mental disorders; HCs, healthy controls; BDI, Beck Depression Inventory; SAS, Self-Rating Anxiety Scale; SBQ-R, Suicide Behaviors Questionnaire-Revised; BSI-C, current suicidal ideation from Beck Scale for Suicidal Ideation; BSI-W, suicidal ideation at one’s worst time from Beck Scale for Suicidal Ideation; SD, standard deviation.

### 2.2. Clinical psychological assessment

#### 2.2.1. Suicide Behaviors Questionnaire-Revised

The Suicide Behaviors Questionnaire-Revised (SBQ-R) includes four items and is used to assess suicidal intention and behaviors in clinical and non-clinical applications (45). Each item has a different score for its options. The total score of the scale ranges from 3 to 18, with higher scores indicating a greater risk of suicide. The Chinese version of the SBQ-R was used in this study (46), and Cronbach’s α coefficient was 0.85.

#### 2.2.2. Beck Scale for Suicidal Ideation

The Beck Scale for Suicidal Ideation (BSI) is a 19-item inventory, and each item is assessed on a 3-point rating scale from 0 to 2 (47). The intensity of suicidal ideation is measured by items 1–5 for “currently” (BSI-C) or “at one’s worst time” (BSI-W). The higher the score, the stronger the suicidal ideation. This study used the Chinese version of the BSI (48, 49), and Cronbach’s α coefficients for the BSI-C and BSI-W were 0.90 and 0.89, respectively.

#### 2.2.3. Beck Depression Inventory

The severity of depression is measured using the Beck Depression Inventory (BDI), which includes 21 items (50). Each question offers the participants with four answers to choose from (each scoring 0–3 points). The total score of the scale ranges from 0 to 63, and the higher the total score, the more severe the depression. The Chinese version of the BDI was used in this study (51), and Cronbach’s α coefficient was 0.90.

#### 2.2.4. Self-Rating Anxiety Scale

The Self-Rating Anxiety Scale (SAS) is a 20-item self-report inventory measuring the intensity of anxiety (52). Each item is assessed on a 4-point rating scale from 1 to 4, among which the 5th, 9th, 13th, 17th, and 19th items are assessed in reverse. In this study, we used the Chinese version of the SAS (53), and Cronbach’s α coefficient was 0.89. We added the scores of all items to obtain the total score; then, we multiplied the total score by 1.25 and used the whole number to obtain a standard score. The higher the standard score, the more severe the anxiety.

### 2.3. Procedure and stimuli

**Figure 1:**
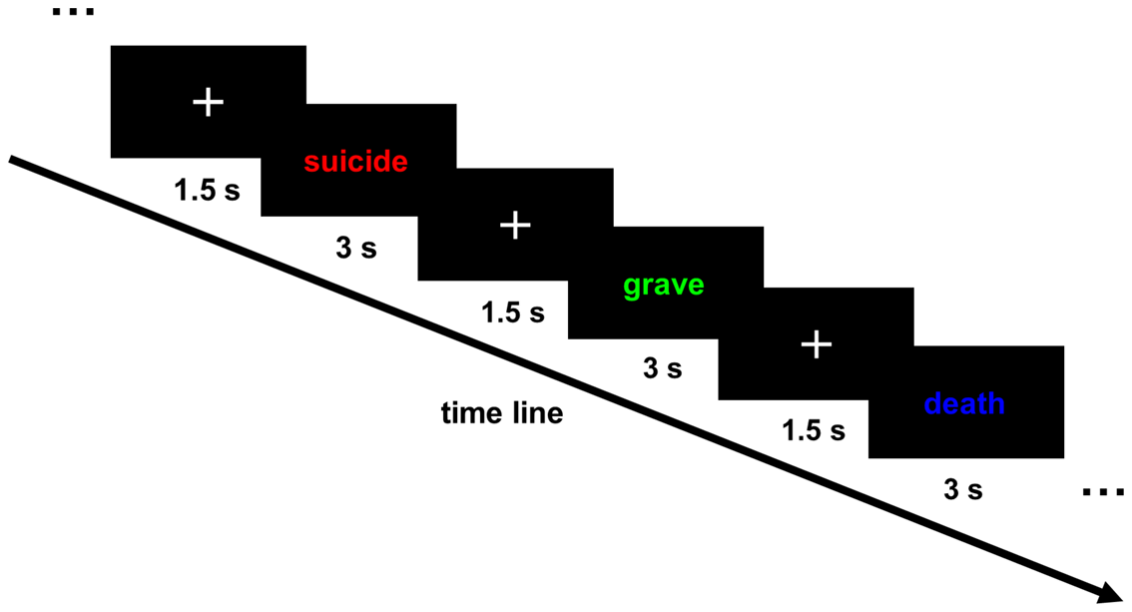
Sequence of trials in the emotional Stroop task. At the beginning of each trial, a fixation cross was displayed at the center of the screen (1.5 s), followed by a target word printed in red, green, or blue ink in a randomized, counterbalanced order. Following the response completion or time limit (3 s), the target word disappeared and the next trial beginning with a fixation cross was initiated.

After completing the questionnaires, the participants were seated comfortably in a chair approximately 60 cm away from a 23.5-inch LCD screen. The participants performed an adaptation of the EST, and they were instructed to ignore the meaning of the target word and respond only to the ink color. The stimulus coding paradigm of the EST was presented using PSYCHTOOLBOX in MATLAB R2021b. The stimulus words were printed in red, green, or blue ink in a randomized, counter-balanced order. The words for each of the four word types (positive, neutral, negative, and suicide) were chosen from the Affective Norms for English Words (54) and additional relevant documents (43, 55), with 10 words in each type. We translated the words into Chinese according to the Modern Chinese Dictionary (see Table 2 for details), resulting in consistent length, concreteness, and comparable frequency of use between the word categories. At the beginning of each trial, a fixation cross was displayed at the center of the screen (1.5 s), followed by a target word presented at the center of the monitor. The target word remained on the screen until the participants responded or the time limit was reached (3 s). Then, the word disappeared and the next trial beginning with a fixation cross was initiated (Figure 1). The participants were instructed to indicate the color of each word as quickly and accurately as possible by pressing color-stickered keys on a keyboard. The “F”, “J”, and “B” keys corresponded to red, green, and blue, respectively. A 12-trial practice session was completed first to ensure that the participants followed the instructions. Following the practice session, they performed a test session that included four separate and counter-balanced blocks corresponding to different word types. Within each block of trials, the order of words was random, and each word was presented in each of the three colors once, for a total of 30 trials. One block lasted for a maximum of 2 min 15 s. There was a 1–2 min break between blocks. To avoid practice effects, the words in the practice session were all neutral words and did not appear in the test session. The overall experiment lasted for approximately 60 min, and the EEG portion lasted for approximately 20 min.

**Table 2:** Positive, neutral, negative, and suicide words (Chinese and English translation) used in the emotional Stroop task.

Positive		Neutral		Negative		Suicide	
幸福	Happy	图书	Book	欺诈	Fraud	出殡	Hold a funeral procession
善良	Kindness	报纸	Paper	白痴	Idiot	丧生	Suffer death
赞扬	Praise	引擎	Engine	耻辱	Shamed	上吊	Hang oneself
惊喜	Surprised	杯子	Glass	谎话	Lie	早逝	Die young
优秀	Excellence	故宫	Forbidden city	骚扰	Harass	服毒	Ingest poison
天真	Innocent	轮胎	Tire	侮辱	Humiliate	埋葬	Burial
成功	Success	纸盒	Carton	嫉妒	Jealousy	死亡	Death
开心	Pleasure	天鹅	Swan	诡计	Trick	坟墓	Grave
快乐	Joy	照片	Photograph	逆境	Adverse circumstance	自杀	Suicide
完美	Perfection	玩具	Toy	难关	Tough obstacle	忌日	Death anniversary

### 2.4. EEG recording and preprocessing

EEG data were acquired by a SynAmps^2^ amplifier and a Scan4.5 acquisition system (both Neuroscan, Charlotte, NC 28269, United States) with standard 64-channel Ag/AgCl electrodes (Neuroscan, Charlotte, NC 28269, United States) placed on the scalp according to the International 10–20 system. The reference electrode was located at the right mastoid, and the ground electrode was placed on the forehead. Inter-electrode impedances were kept below 5 kΩ. EEG data were digitized continuously at a 1,000 Hz sampling rate.

The preprocessing for EEG data was conducted offline using the EEGLAB toolbox (v2021.1) in MATLAB R2021b. EEG raw data were re-referenced to bilateral mastoid and band-pass filtered between 0.1 and 30 Hz (24 dB/octave slope), followed by down-sampling to 200 Hz. Independent component analysis was employed to identify and remove eye movements, blinks, and muscle-related artifacts. EEG data were segmented into 1,200 ms epochs beginning 200 ms before word onset (with word onset as time 0). We aligned all trials to a common baseline for each channel by subtracting the average of the 200 ms pre-stimulus interval from each trial’s waveform. The epochs in which EEG voltage exceeded ±100 μV relative to the baseline were discarded. Approximately 29 trials remained in each condition (positive, neutral, negative, and suicide). The epochs of each word type were averaged separately per participant at each electrode site.

### 2.5. ERP analyses

All ERPs were initially identified using the grand average (the average of each group of participants) at site Pz, where ERPs tend to obtain high signal to noise ratio (SNR). The EPN and P300 were observed in every group across all word types, with the EPN appearing at approximately 200 ms and the P300 appearing at approximately 360 ms. Based on previous studies (23), the EPN amplitude was quantified using the mean of all the data points within ±25 ms of the peak amplitude, and its latency was identified using the peak latency in the latency window of 150–280 ms for each participant. Because the P300 tends to occupy a wide latency window, its amplitude was quantified using the mean of all the data points in the latency window of 250–500 ms. P300 latency was identified using the 50% area latency measurement, that is, the time point at which the area under the ERP curve within a certain time window can be divided equally.

Electrode sites were grouped into regions of interest (ROIs) based on where the ERP components have been quantified in previous studies (23, 32, 56–61). The ROIs for the EPN were at parieto-occipital sites, whereas the ROIs for the P300 were at centro-parietal sites. Therefore, the EPN was measured at parietal (P3, Pz, P4), parieto-occipital (PO3, POz, PO4), and occipital (O1, Oz, O2) clusters, and the P300 was quantified at central (C1, Cz, C2), centro-parietal (CP1, CPz, CP2), and parietal (P1, Pz, P2) clusters.

### 2.6. Statistical analyses

Demographic and clinical psychological characteristics among groups were compared using one-way analysis of variance (ANOVA). The Chi-square test was used for sex comparison. We used the Kruskal-Wallis H test when data showed a non-normal distribution. Behavioral performance data (response accuracy and reaction time) and ERP features (amplitude and latency) were analyzed separately using a mixed-model ANOVA with group (HC, SINMD, SIMD) as a between-subject factor and word type (positive, neutral, negative, and suicide) as a within-subject factor. A generalized estimating equation (GEE) was used when data did not satisfy the normal distribution or homogeneity of variance assumptions. For violations of the sphericity assumption, the Greenhouse–Geisser correction was applied to correct the degrees of freedom. Simple effects were explored when decomposing significant interactions, and Bonferroni corrected probability values (*p*-values) were used for post-hoc comparisons. We further performed Spearman correlation analyses between ERP characteristics and clinical psychological variables to identify risk factors associated with suicide. Bonferroni corrections for multiple comparisons were applied for the correlations. All the statistical analyses were performed with SPSS 26.0. The alpha level of significance was set at 0.05.

## 3. Results

### 3.1. Demographic and clinical psychological characteristics

The participants in this study consisted of undergraduates and postgraduates. The MINI found that the main mental disorders of the sample were depression and anxiety. Therefore, we calculated the severity of depression and anxiety to evaluate the level of mental disorders in the sample. The demographic and clinical psychological characteristics of the HC, SINMD, and SIMD groups are presented in Table 1. There were no significant differences in sex or age across groups (*p* > 0.05 in all cases). A one-way ANOVA revealed significant differences in SAS across groups (*F* = 4.292, *p* = 0.025). A Kruskal-Wallis H test showed that the scores of other clinical psychological scales significantly differed among groups (BDI: *H* = 12.138, *p* = 0.002; SBQ-R: *H* = 29.483, *p* < 0.001; BSI-C: *H* = 10.168, *p* = 0.006; BSI-W: *H* = 28.315, *p* < 0.001). Post-hoc comparisons revealed that the SIMD group had significantly larger scores of BDI, SAS, SBQ-R, BSI-C, and BSI-W than the HC group (BDI: *p* = 0.002; SAS: *p* = 0.007; SBQ-R: *p* < 0.001; BSI-C: *p* = 0.013; BSI-W: *p* < 0.001) and significantly larger SAS and BSI-C scores than the SINMD group (SAS: *p* = 0.036; BSI-C: *p* = 0.022). The SBQ-R and BSI-W scores of the SINMD group were also greater than those of the HC group (both *p* < 0.001). The results demonstrated that the SIMD group had more severe depression, anxiety, suicide risk, and suicidal ideation compared to the HC group. There was a significant difference in suicide risk and suicidal ideation between the SINMD and HC groups, but not in depression and anxiety. The significant differences of clinical psychological characteristics between the SIMD and SINMD groups were shown in the levels of anxiety and suicidal ideation.

### 3.2. Behavioral results

**Figure 2:**
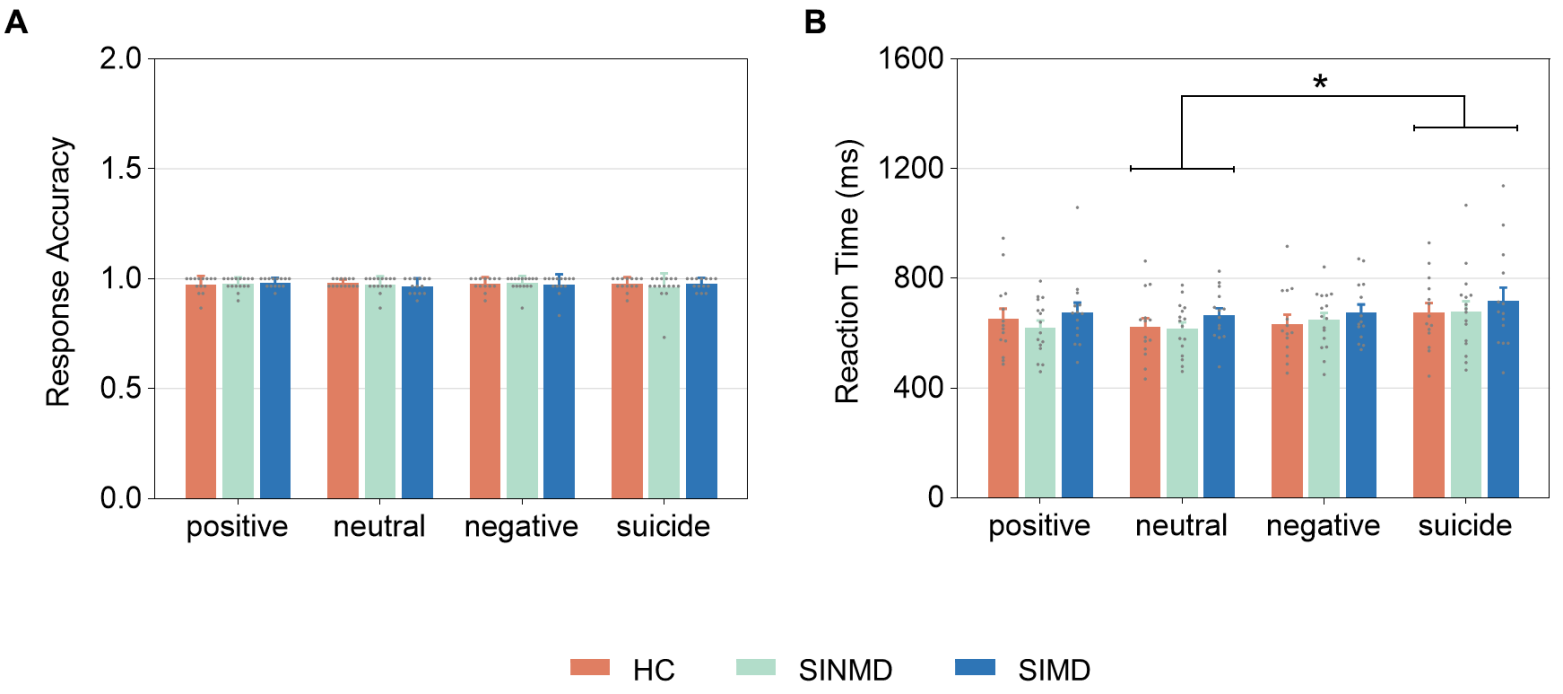
Behavioral performance on the emotional Stroop task. **(A)** Response accuracy. **(B)** Reaction time. Error bars represent standard deviation of the mean. The gray, solid points reflect individual participant data. Asterisk (*) depicts significant differences (*p* < 0.05). SIMD, individuals with suicidal ideation as well as mental disorders; SINMD, individuals with suicidal ideation but no mental disorders; HC, healthy controls.

Response accuracy and reaction time are presented in Figure 2. The average response accuracy values for each word type were above 0.9 in each group. Further analysis showed that response accuracy was not significantly different across groups, Wald χ^2^ (2) = 0.137, *p* = 0.934. Similarly, there was no significant effect of word type, Wald χ^2^ (3) = 1.622, *p* = 0.654, or interaction between group and word type, Wald χ^2^ (6) = 6.826, *p* = 0.337.

For reaction time, an ANOVA showed that the main effect of group failed to reach significance, *F*(2, 41) = 0.639, *p* = 0.533, $\eta_{p}^{2}$ = 0.030. There was a significant effect of word type, *F*(3, 123) = 4.992, *p* = 0.006, $\eta_{p}^{2}$ = 0.109. Post-hoc comparisons revealed that reaction time was significantly longer for suicide words compared to neutral words (*p* = 0.017). There were no other significant differences across word types (*p* > 0.05 in all cases). The interaction between group and word type was not significant, *F*(6, 123) = 0.324, *p* = 0.886, $\eta_{p}^{2}$ = 0.016.

### 3.3. ERP results

**Figure 3:**
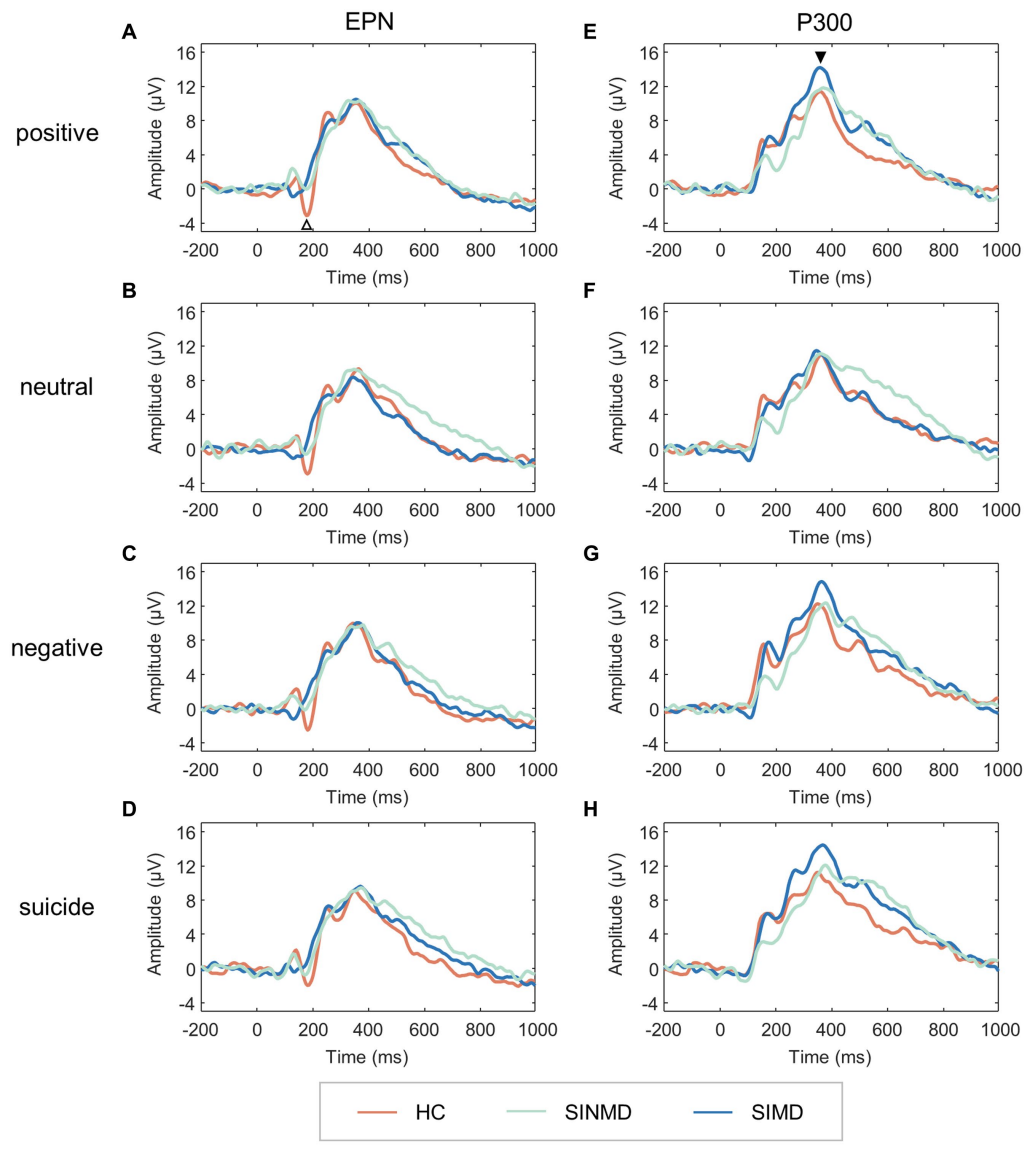
Grand average ERPs. Left: ERP courses of the ROIs for EPN following **(A)** positive, **(B)** neutral, **(C)** negative, and **(D)** suicide words. The white upward arrow reflects the EPN. Right: ERP courses of the ROIs for P300 following **(E)** positive, **(F)** neutral, **(G)** negative, and **(H)** suicide words. The black downward arrow reflects the P300. SIMD, individuals with suicidal ideation as well as mental disorders; SINMD, individuals with suicidal ideation but no mental disorders; HC, healthy controls; EPN, early posterior negativity.

**Figure 4:**
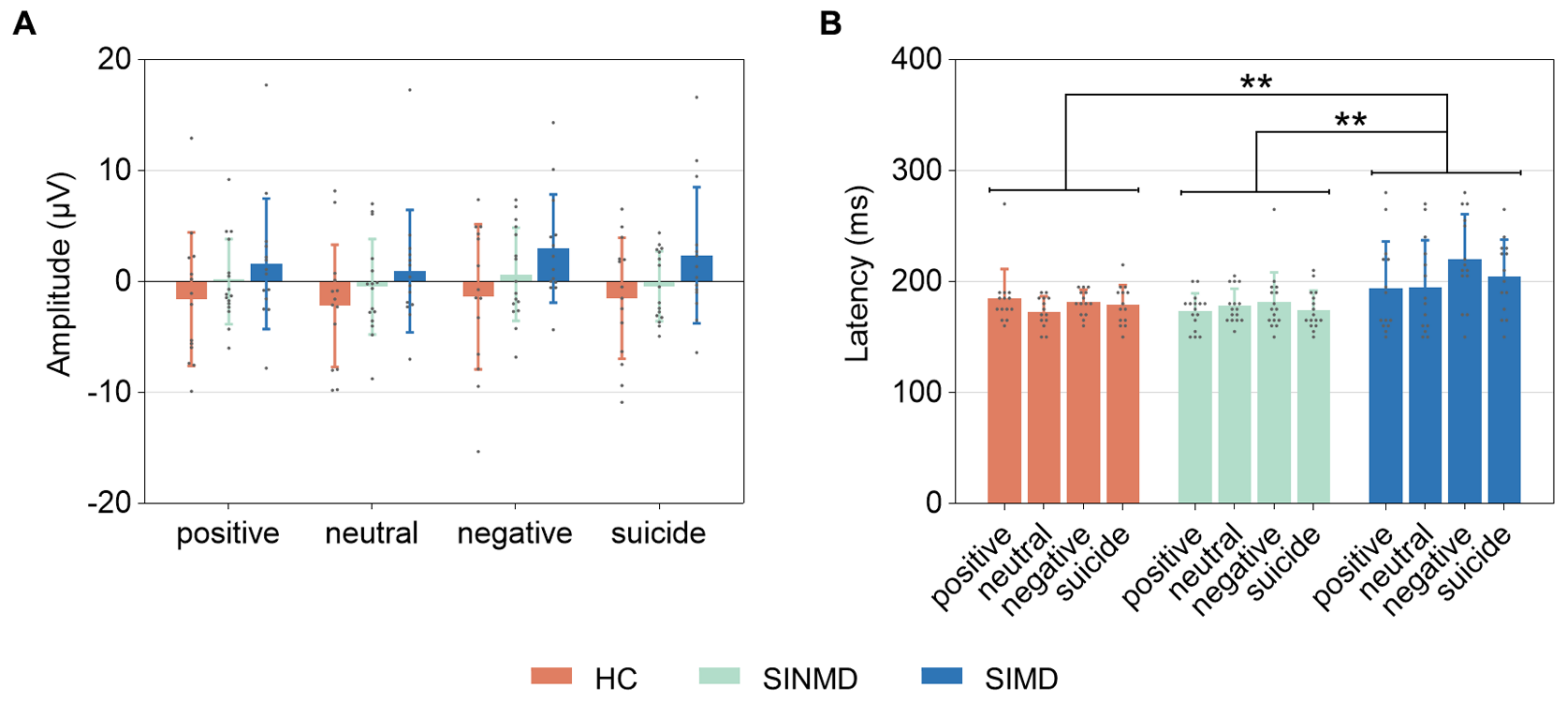
EPN characteristics quantified at parieto-occipital sites. **(A)** EPN amplitude. **(B)** EPN latency. Error bars represent standard deviation of the mean. The gray, solid points reflect individual participant data. Asterisks (**) depict significant differences (*p* < 0.01). SIMD, individuals with suicidal ideation as well as mental disorders; SINMD, individuals with suicidal ideation but no mental disorders; HC, healthy controls.

Figure 3 shows the grand average ERPs. The EPN and P300 were observed in three groups across all word types, with the EPN appearing at approximately 200 ms (white arrow) and the P300 appearing at approximately 360 ms (black arrow). As depicted in Figures 4 5, the amplitude and latency values of the ERP components were quantified at their ROIs.

**Figure 5:**
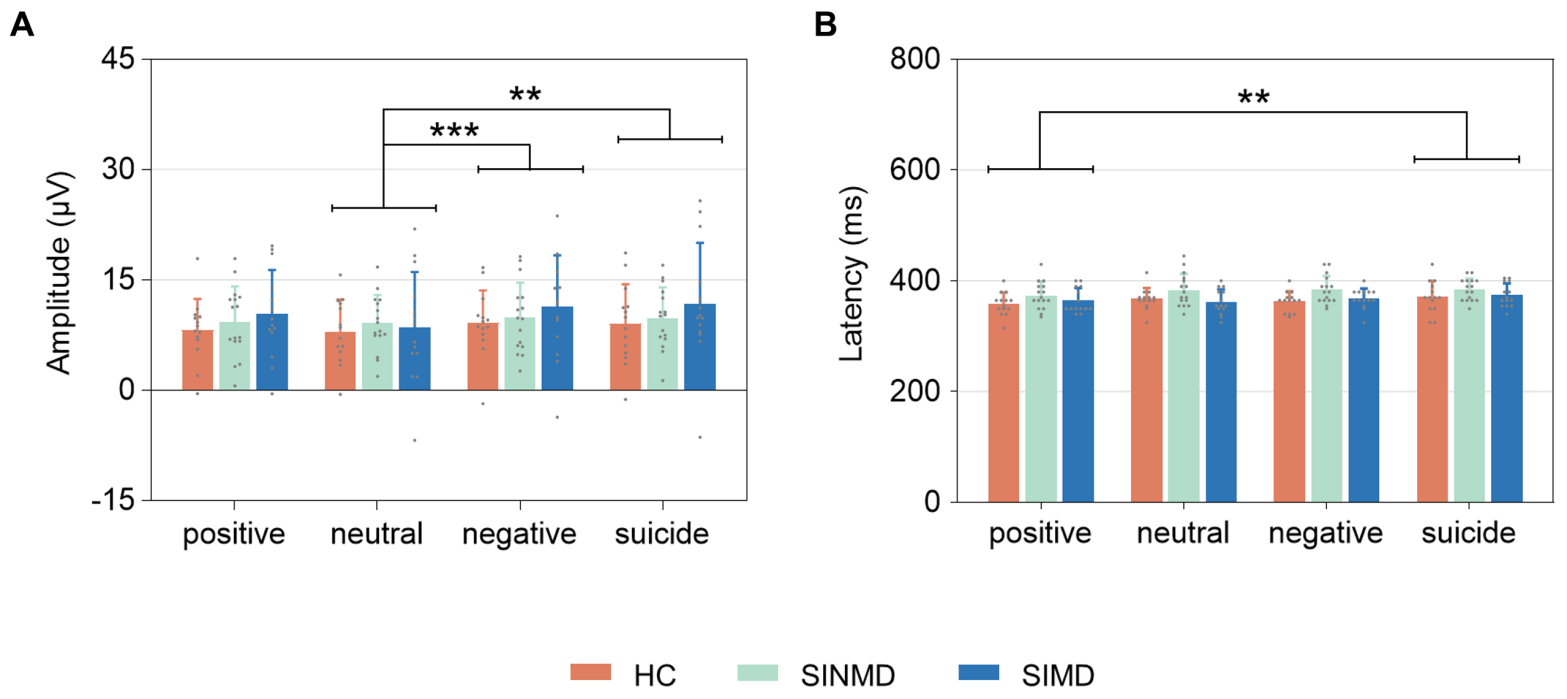
P300 characteristics quantified at centro-parietal sites. **(A)** P300 amplitude. **(B)** P300 latency. Error bars represent standard deviation of the mean. The gray, solid points reflect individual participant data. Asterisks (**) and (***) depict significant differences (*p* < 0.01, *p* < 0.001, respectively). SIMD, individuals with suicidal ideation as well as mental disorders; SINMD, individuals with suicidal ideation but no mental disorders; HC, healthy controls.

#### 3.3.1. EPN

For EPN amplitude, an ANOVA revealed that there was no significant effect of group, *F*(2, 41) = 2.068, *p* = 0.139, $\eta_{p}^{2}$ = 0.092, or word type, *F*(3, 123) = 2.324, *p* = 0.078, $\eta_{p}^{2}$ = 0.054. The interaction between group and word type was also not significant, *F*(6, 123) = 0.385, *p* = 0.888, $\eta_{p}^{2}$ = 0.018.

For EPN latency, a GEE analysis showed a significant effect of group, Wald χ^2^ (2) = 12.299, *p* = 0.002. Post-hoc comparisons indicated that the EPN latency was significantly longer in the SIMD group than in the HC group (*p* = 0.007) and the SINMD group (*p* = 0.001). There was also a significant effect of word type, Wald χ^2^ (3) = 10.186, *p* = 0.017, with earlier EPN latency to neutral words compared to negative words (*p* = 0.049). The interaction between group and word type was not significant, Wald χ^2^ (6) = 10.937, *p* = 0.090.

#### 3.3.2. P300

For P300 amplitude, there was no significant difference across groups, *F*(2, 41) = 0.484, *p* = 0.620, $\eta_{p}^{2}$ = 0.023. The main effect of word type was significant, *F*(3, 123) = 6.430, *p* < 0.001, $\eta_{p}^{2}$ = 0.136. Post-hoc comparisons indicated that P300 amplitude was greater to negative (*p* = 0.001) and suicide (*p* = 0.002) words than to neutral words. There was no significant interaction between group and word type, *F*(6, 123) = 1.245, *p* = 0.288, $\eta_{p}^{2}$ = 0.057.

For P300 latency, an ANOVA revealed that latency was not significantly different across groups, *F*(2, 41) = 2.893, *p* = 0.067, $\eta_{p}^{2}$ = 0.124. The main effect of word type was significant, *F*(3, 123) = 5.570, *p* = 0.001, $\eta_{p}^{2}$ = 0.120. Post-hoc comparisons showed that P300 latency was significantly earlier to positive words compared to suicide words (*p* = 0.001). There was no significant interaction between group and word type, *F*(6, 123) = 1.072, *p* = 0.383, $\eta_{p}^{2}$ = 0.050.

### 3.4. Correlations

We found between-group differences in EPN latency, so the correlation analyses were first conducted between scores of clinical psychological scales (assessing the severity of mental disorders and suicidal ideation) and EPN latencies of the four word types for individuals with suicidal ideation. As depicted in Table 3, the pooled correlation showed a positive correlation between EPN latency for neutral words and BSI-C score (*r* = 0.423, Bonferroni-*p* = 0.049 < 0.05). However, the correlations measured within the groups were not significant (SINMD and SIMD).

**Table 3:** Correlation coefficients between the scores of clinical psychological scales and the EPN latency of each word type for individuals with suicidal ideation (*r*).

Clinical scales	EPN latency			
	Positive	Neutral	Negative	Suicide
SINMD+SIMD				
BDI score	0.201	0.048	0.366	0.131
SAS score	0.228	0.035	0.310	0.222
SBQ-R score	0.207	0.223	0.155	0.369
BSI-C score	0.242	0.423*	0.260	0.388
BSI-W score	0.033	0.027	−0.091	0.329
SINMD				
BDI score	−0.329	−0.209	−0.108	−0.376
SAS score	−0.445	0.295	−0.192	0.218
SBQ-R score	−0.155	0.373	−0.085	0.024
BSI-C score	0.005	−0.473	−0.223	−0.232
BSI-W score	0.228	0.393	−0.037	0.133
SIMD				
BDI score	0.188	−0.063	0.328	−0.037
SAS score	0.246	−0.169	0.272	−0.037
SBQ-R score	0.413	0.080	−0.049	0.270
BSI-C score	0.116	0.426	0.072	0.261
BSI-W score	−0.165	−0.198	−0.426	0.268

*p < 0.05. SIMD, individuals with suicidal ideation as well as mental disorders; SINMD, individuals with suicidal ideation but no mental disorders; EPN, early posterior negativity; BDI, Beck Depression Inventory; SAS, Self-Rating Anxiety Scale; SBQ-R, Suicide Behaviors Questionnaire-Revised; BSI-C, current suicidal ideation from Beck Scale for Suicidal Ideation; BSI-W, suicidal ideation at one’s worst time from Beck Scale for Suicidal Ideation.

We also analyzed the correlations between scores of clinical psychological scales (assessing the severity of mental disorders and suicidal ideation) and other ERP signatures of the four word types for individuals with suicidal ideation. As depicted in Table 4, P300 latency for positive words was positively correlated with BSI-C score (*r* = 0.690, Bonferroni-*p* = 0.008 < 0.01) in the SINMD group. The higher the BSI-C score, the longer the P300 latency for positive words. Correlations for the other ERP signatures are not shown as they were not significant.

**Table 4:** Correlation coefficients between the scores of clinical psychological scales and the P300 latency of each word type for individuals with suicidal ideation (*r*).

Clinical scales	P300 latency			
	Positive	Neutral	Negative	Suicide
SINMD+SIMD				
BDI score	−0.032	−0.011	−0.159	0.034
SAS score	0.008	−0.110	−0.119	0.010
SBQ-R score	0.067	−0.064	−0.147	0.051
BSI-C score	0.223	0.079	−0.166	0.247
BSI-W score	0.112	0.104	0.066	0.003
SINMD				
BDI score	0.093	0.400	0.349	0.210
SAS score	0.082	0.076	0.355	0.218
SBQ-R score	−0.151	−0.090	−0.131	−0.150
BSI-C score	0.690**	0.541	0.430	0.353
BSI-W score	−0.119	−0.028	−0.070	−0.132
SIMD				
BDI score	0.038	−0.007	−0.414	0.149
SAS score	0.108	0.032	−0.261	0.073
SBQ-R score	0.377	0.266	0.087	0.351
BSI-C score	0.383	0.378	−0.155	0.535
BSI-W score	0.462	0.553	0.511	0.248

**p < 0.01. SIMD, individuals with suicidal ideation as well as mental disorders; SINMD, individuals with suicidal ideation but no mental disorders; BDI, Beck Depression Inventory; SAS, Self-Rating Anxiety Scale; SBQ-R, Suicide Behaviors Questionnaire-Revised; BSI-C, current suicidal ideation from Beck Scale for Suicidal Ideation; BSI-W, suicidal ideation at one’s worst time from Beck Scale for Suicidal Ideation.

## 4. Discussion

The present study explored the behavioral responses and electrophysiological bases of attentional bias to emotional stimuli in individuals with suicidal ideation during a modified EST. Response accuracy to word color among groups was not affected by word type, which is consistent with previous literature (23). There was an emotional Stroop effect for reaction time such that reaction times were significantly slower for suicide words than for neutral words. However, the results are inconsistent with previous findings that show significantly longer response times in suicide attempters for suicide words and no differences for HCs (23, 24). The samples in previous studies were clinical suicide attempters, whereas this study analyzed non-clinical individuals with suicidal ideation. The discrepancies between the present results and those of previous studies may be attributable to the differences in participant populations, suggesting that early identification of suicidal ideation requires a more sensitive method than behavioral measures.

Two ERP components (EPN and P300) were examined to reveal group differences in attention processing. The EPN component represents early attentional selection and automatic emotional processing (27–31), which is related to the processing of semantic information (e.g., positive, neutral, negative, and suicide words). In this study, the SIMD group exhibited longer EPN latency compared to the SINMD and HC groups, suggesting that the early attention processing of the SIMD group was affected during the EST, and the automatic and rapid processing of emotional information decreased. However, between-group differences in EPN across all word types did not support the hypothesis of attentional bias in the SINMD group compared to the HC group, as there was no significant difference in EPN. One possible reason for this is that in our study, we used suicidal ideation at one’s worst time, but not current suicidal ideation, to meet the requirements (i.e., significantly more severe suicidal ideation in SIMDs and SINMDs than in HCs and no significant difference in suicidal ideation between SIMDs and SINMDs). Current suicidal ideation was slightly higher (but not significantly) in the SINMD group than in the HC group and significantly lower in the SINMD group than in the SIMD group. Therefore, we speculate that the slightly higher current suicidal ideation in SINMDs did not significantly affect the early stage of attention processing in the context of the EST. Furthermore, the SIMD group differed from the SINMD and HC groups in terms of mental disorders and current suicidal ideation. Therefore, the influence of mental disorders on the prolonged EPN latency in the SIMD group cannot be ruled out. The P300 component is related to attention and reflects the speed and efficiency of processing during stimulus evaluation. P300 amplitude was greater for negative and suicide words than for neutral words, which is consistent with previous studies (32, 41, 42), indicating enhanced processing of (or an attentional bias toward) negative information. We did not find a difference in P300 in the SINMD or SIMD groups, suggesting that there was no difference in attentional resource distribution and stimuli assessment during the conflict between word-reading and color-naming.

Correlation analyses were conducted to explore the relationships between clinical variables and ERP characteristics. Our results showed that the prolonged EPN latencies to neutral words were positively correlated with BSI-C scores in individuals with suicidal ideation. However, the correlations measured within the groups (SINMD and SIMD) were not significant. Therefore, the pooled correlation between EPN latency for neutral words and BSI-C score resulted from the heterogeneity of groups. In the SINMD group, P300 latency for positive words was positively correlated with BSI-C score. These results may reflect that delayed responses or additional processing to positive information may lead individuals with suicidal ideation to an incorrect interpretation of external events. Overall, our findings contribute to a more comprehensive understanding of the factors influencing suicide behavior.

The present study has several limitations to consider. First, due to the limited sample size, the current study is underpowered. The findings need to be further validated, preferably with a larger sample size. Second, the number, or ratio, of word attributes (such as adjectives:nouns:verbs) was not consistent across positive, neutral, negative, and suicide words. Different mental disorders lead to inherent heterogeneity in the SIMD group. It cannot be ruled out that different mental disorders may lead to opposite trends in neural responses, which may cancel out, reducing the overall effect. Future studies should strictly control the experimental conditions, using a standardized approach when exploring the attentional bias of individuals with suicidal ideation. Third, this study enrolled individuals with lifetime suicidal ideation, rather than those with current suicidal ideation, resulting in BSI-C differences between the SIMD and SINMD groups, but not between the SINMD and HC groups. Therefore, our findings do not allow ascribing group differences to the presence or absence of mental disorders. Subsequent studies should keep current suicidal ideation comparable across groups to make the conclusions more robust. Finally, future research can broaden the study sample by including suicide attempters. In addition, analyses of frequency and time-frequency domain may be helpful to obtain a more integrated understanding.

## 5. Conclusion

To the best of our knowledge, this is the first study to assess attentional bias to emotional stimuli in a non-clinical sample of university students with suicidal ideation by recording ERPs during the EST. In summary, we explored the possible suicide-related neurophysiological indicators during the EST, and our results suggest that the neural characteristics in the SIMD group differ from those in the SINMD and HC groups. The present findings offer a new understanding of the neural mechanism of attention processing in individuals with suicidal ideation and provide a reference for subsequent research and clinical suicide prevention.

## Data availability statement

The datasets presented in this article are not readily available because we promised participants that their data would not be disclosed in the informed consent form, even if they were anonymous and unidentifiable. Requests to access the datasets should be directed to SL, shuangliu@tju.edu.cn.

## Ethics statement

The studies involving humans were approved by the Ethics Committee of Tianjin University, China. The studies were conducted in accordance with the local legislation and institutional requirements. The participants provided their written informed consent to participate in this study.

## Author contributions

YS and MD contributed to the rationale as well as the design of the study and recruited the participants. MD gained ethical approval. YS collected and analyzed the EEG data. The manuscript was written by YS. All authors contributed to the article and approved the submitted version.

## Funding

This study was supported by the National Natural Science Foundation of China (Grant ID: 81925020).

## Conflict of interest

The authors declare that the research was conducted in the absence of any commercial or financial relationships that could be construed as a potential conflict of interest.

## Publisher’s note

All claims expressed in this article are solely those of the authors and do not necessarily represent those of their affiliated organizations, or those of the publisher, the editors and the reviewers. Any product that may be evaluated in this article, or claim that may be made by its manufacturer, is not guaranteed or endorsed by the publisher.

## Abbreviations

SIMD: individuals with suicidal ideation as well as mental disorders
SINMD: individuals with suicidal ideation but no mental disorders
BSI-C: current suicidal ideation from Beck Scale for Suicidal Ideation
BSI-W: suicidal ideation at one’s worst time from Beck Scale for Suicidal Ideation
